# The impact of a skipped-generation family structure on early child development in Khon Kaen Province: a prospective cohort study

**DOI:** 10.1038/s41598-023-44238-9

**Published:** 2023-10-06

**Authors:** Piyanan Photichai, Varisara Luvira

**Affiliations:** Department of Community Medicine, Faculty of Medicine, Khon Kaen University, Khon Kaen, 40002 Thailand

**Keywords:** Health care, Psychology and behaviour

## Abstract

Parenting in early childhood is related to child development. This study aimed to investigate the impact of a skipped-generation family structure on early child development. This prospective cohort study collected data on children aged 6 to 18 months from 132 non-skipped-generation families and 115 skipped-generation families from primary care units in Khon Kaen province for 1 year. The data were collected using a structured questionnaire administered through face-to-face interviews, as well as the Denver II instrument for assessing child development. Using multivariate logistic regression, the impact of a skipped-generation family structure on infant development was analysed, and adjusted relative risks (aRRs) are presented. We found that 19.83% (49/247) of the children had suspected delayed development in all domains. Most of these children resided in skipped-generation families, accounting for 27.82% of the sample (32/115). After adjusting for other factors, it was found that male children from skipped-generation families had a higher risk of suspected delayed language development (aRR = 14.56, 95% CI = 1.34 to 158.34, *p* = 0.028). In conclusion, the parental practices of skipped-generation families are suspected of causing delayed language development in boys. Models of early childhood development should be established for skipped-generation families.

## Introduction

Growth in psychomotor ability is defined as development^1^. The process of development is intricate, commencing during the prenatal stage and extending throughout the duration of adulthood^2^. Early childhood development, which consists of cognitive, motor, language, and social-emotional development^3^, influences early childhood experiences, which in turn affects lifelong health, well-being, and productivity^4^. Child development is influenced by both genetics and the environment^5^; the environment in particular impacts the structure and function of children's brains^6^, which in turn influences their overall development.

Generally, developmental delay is diagnosed when a child fails to reach developmental milestones at the same rate as their peers from the same population^7^. At least 250 million children worldwide from low-income and middle-income countries are at risk of developmental delay^8,9^. As a result of poverty, health creation, care provision, learning promotion, nutrition, environmental contaminants, and stress issues are problematic^10–13^. Socioeconomic factors and family factors are significant environmental influences that impact child development^1^. Suboptimal child outcomes are often linked to low-income families due to the inadequacy of their home environments in fostering optimal child development^9^. The impact of parenting on a child's development is significant. Insufficient knowledge or a strained caregiver–child relationship can impede a child's development by influencing the type of parenting they receive^14–17^. Malnutrition can lead to various deficiencies in children, including iron deficiency and zinc deficiency, which can have detrimental effects on child development^1^.

The influence of caregivers on early infant development is of considerable importance. Generally, the mother serves as the principal caregiver, while the father serves as the household's chief. Because of this, a mother's care is essential for nurturing her children^8^. Nevertheless, the dynamic and ongoing transformations in the economy and society, coupled with the unequal distribution of wealth, result in some children being exposed to the phenomenon of parental migration, resulting in skipped-generation families in which there is no parent generation but only grandchild, grandparent, and/or great-grandparent generations who live together. Therefore, in these families, elderly grandparents are primarily responsible for providing care to children^18,19^. Children who have been left behind may have an increased likelihood of experiencing developmental delays^18,20,21^. This situation could potentially arise as a result of elderly grandparents assuming the role of primary caregivers; they may encounter limitations due to a lack of comprehension of the significance of positive parenting practices and cognitive stimulation, such as reading to the child or encouraging play. This results in children being left to play alone or watch television, which has a deleterious impact on their development^22^, as well as the health of their elderly grandparents—i.e., their caregivers—which deteriorates with age^23^. However, most families practice parental migration due to family financial issues, which leads to a better financial status for the family^24^. This proves to have positive effects on the health, nutrition, and educational outcomes of children^25^, which may positively influence their development.

It was estimated that the number of infants left behind worldwide was in the hundreds of millions^26^. This issue is of major concern in the Asian region. A significant proportion of children residing in rural areas of China are affected by the phenomenon of being "left behind" by one or both of their migrant parents^26,27^. Existing research on the impact of a skipped-generation family structure on early childhood development has yielded inconclusive findings. A meta-analytic review conducted in 2015 revealed the presence of publication bias in studies that reported less favorable development outcomes for migrant and left-behind children^28^. A subsequent survey conducted in China revealed that rural children who were left behind by both parents experienced a more pronounced delay in cognitive development compared to rural children who resided with both parents^22^. Another study, which was a longitudinal survey that followed children from the age of 6 months to the age of 30 months, found that maternal migration had a negative effect on cognitive development. Specifically, when migration occurred before children reached the age of 12 months, a reduction in cognitive development scores by approximately 0.3 standard deviations by the age of 2 years was observed. Potential mechanisms contributing to developmental delays in children may arise from factors such as diminished dietary diversity and limited participation in stimulating activities within skipped-generation families^21^. This is related to a study conducted in Thailand, which revealed that the absence of a mother in the family increased the risk of developmental delays in early childhood^18^. However, an additional study, which was also conducted in a rural region of China, yielded slightly divergent findings regarding early childhood development. Parental migration did not correlate with a child's cognitive, motor, or social-emotional development; however, it was associated with delayed language development in early childhood^20^. Recently, a prospective cohort study was conducted on Chinese children to evaluate the correlation between parental migration and various indicators of early childhood development. The study revealed a negative correlation between parental migration and both cognitive stimulation and the quality of the home environment. The strength of the associations was heightened when both parents had migrated^29^.

Thailand is a country with a high rate of parental migration, with over one in five children being left behind^30^. This phenomenon is particularly prominent in the northern and northeastern regions of the country, primarily attributed to prevailing poverty conditions^31^. Khon Kaen is a province located in the northeastern region of Thailand, which has a significant concentration of labour migration. A survey was conducted in the province, and the findings indicated the presence of developmental delay in early childhood, with minimal or no observed tendency towards improvement. Furthermore, the available data on the impact of a skipped-generation family structure on early childhood development are inconclusive based on previous research. Accordingly, the researchers were interested in determining whether a skipped-generation family structure in which children are raised by grandparents has an impact on child development in comparison to a nuclear or extended family structure in which children are raised by their father and/or mother. This research examined a cohort of normally developing infants at 6 months of age and followed them longitudinally for a period of 1 year. Development was measured using Denver II tools, as 18-month-old children exhibit development in several aspects. The outcomes of such development are influenced in part by parenting, which could be improved in the case of delayed development. This study could be used as planning guidelines for child and family support. Moreover, the data from this study could be utilized in policy formulation and the examination of development strategies to ensure a more equitable distribution of economic opportunities among communities. Ultimately, these factors could reduce the need for parental migration during parenthood and early childhood development. Thus, appropriate nurturing could be provided for children.

## Methods

### Study design

This prospective cohort study aimed to monitor early child development in children raised by their grandparents and/or great-grandparents in skipped-generation families compared to children raised by their father and/or mother in non-skipped-generation families. The purpose of the 1-year monitoring period was to examine child development across various primary caregiver and family types, with the aim of comparing the presence of appropriate or delayed development.

### Operational definition

In this study, a skipped-generation family was defined as a family that included no parent generation but only grandchild, grandparent, and/or great-grandparent generations who lived together, with no other individuals or relatives. In addition, a 6-month-old infant being cared for by a grandfather (paternal or maternal), grandmother (paternal or maternal), or great-grandparent for at least 2 months during early childhood was defined as a skipped-generation family. The definitions were as follows:

1. In the household, apart from the grandfather (paternal or maternal), grandmother (paternal or maternal), or great-grandparent, there were no other individuals or relatives.
2. The grandfather (paternal or maternal), grandmother (paternal or maternal), or great-grandparents were the child's primary caregivers for at least 2 months.
3. The father and mother of the 6-month-old child had not been with the child for more than 2 months due to migration for work in other provinces or abroad.
4. The father and/or mother of the 6-month-old child contacted the grandfather (paternal or maternal), grandmother (paternal or maternal), or great-grandparents, as well as the child, through all available channels of communication. The father and mother had visited the child once per week, once per month, occasionally due to festival seasons, or not at all after migration.

In this study, a nuclear family was defined as a family with members from 2 generations, i.e., the father and/or mother and child, with no additional individuals or relatives residing in the household.

In this study, an extended family was defined as a family with members from three generations, i.e., a grandparent, great-grandparent, parent, and child.

### Setting and participants

The data were collected from caregivers and 6-month-old children in early childhood who visited primary care units in three districts of Khon Kaen Province, namely, Mueang Khon Kaen District, Ban Phai District, and Ban Haet District, to receive vaccinations for the children. The three districts in Khon Kaen Province are considered to be among the largest, based on available data that indicate significant levels of population migration occurring within these regions.

The target population was as follows: (1) primary caregivers who had cared for 6-month-old children for at least 2 months and who resided with the children. These caregivers were divided into 2 groups: a group of grandparents or great-grandparents in skipped-generation families and a group of fathers and/or mothers in nuclear and extended families. (2) 6-month-old children with both types of family structure.

Regarding families who participated in this study, we considered both children and their caregivers who met the inclusion and exclusion criteria. For children, we included only those born full-term with no genetic or development-affecting diseases who had normal development as determined by Denver II tools. For caregivers, we included those who were at least 18 years old and able to communicate, comprehend, and respond to questions. Children who were unable to undergo the Denver II developmental assessment due to illnesses or other conditions were excluded from the study, and we excluded caregivers who were unable to participate in the interview due to illnesses or other conditions.

The data collection process was initiated in February 2021 and was followed by ongoing monitoring for a duration of 1 year. The data collected was analysed to assess child development at 18 months of age using Denver II tools.

### Sample size calculation

The sample size was calculated using a formula for cohort studies^32^. The proportion of children in the exposed group (skipped-generation families) with delayed was P1 = 34%. The proportion of children in the unexposed group (nuclear or extended families) with delayed development was P2 = 17%. The ratio between the two groups was 1:1, with an α of 5%, a power of 80%, and a Zα/2 value of 1.96. The P1 and P2 values were obtained from a previous study conducted in Thailand^18,33^. The calculated sample size for each group was 137 individuals. The sample size for this study was adjusted to account for a potential loss of 5% of the participants. Consequently, the total sample size per group was 145 individuals.

**Figure 1:**
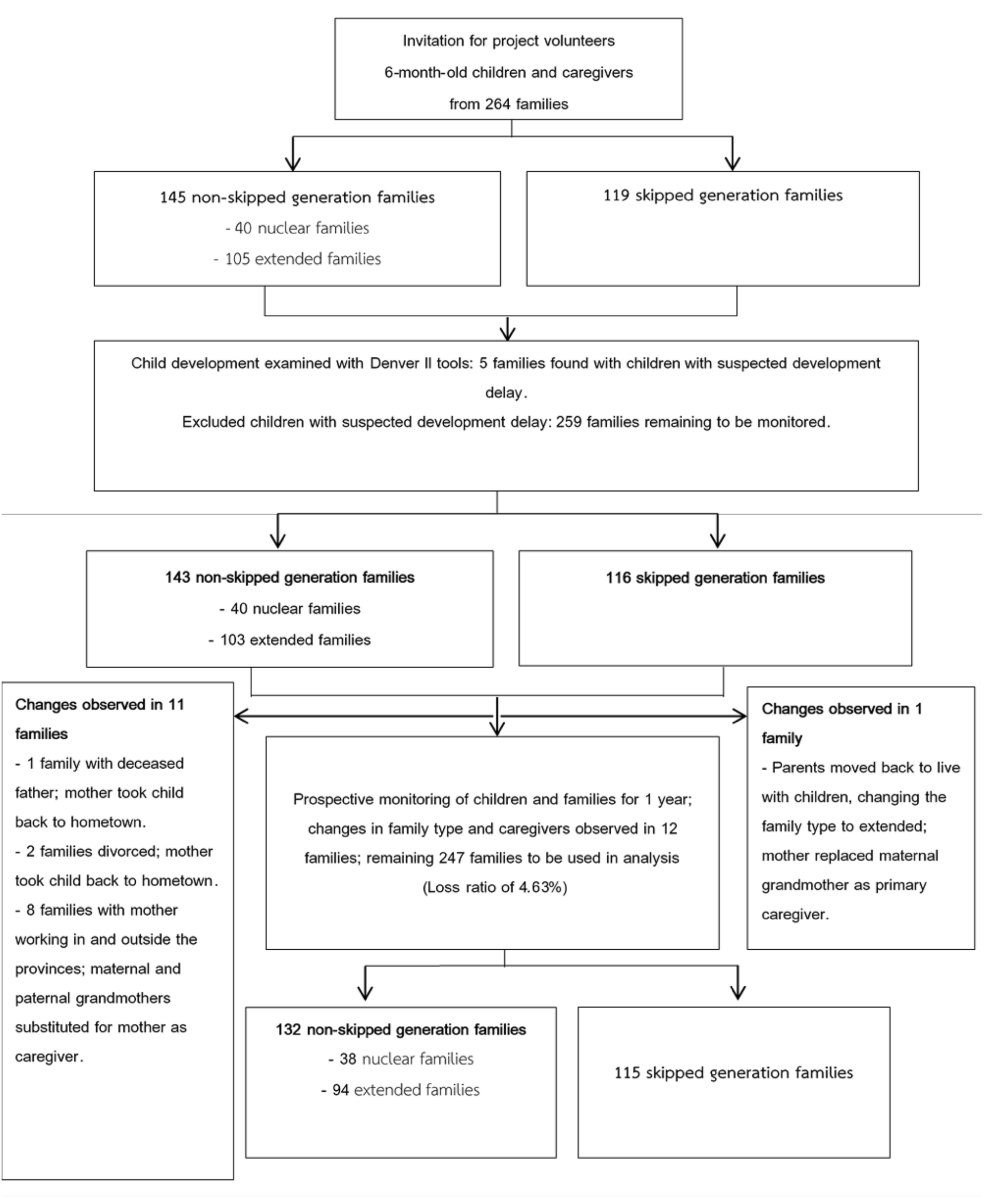
Flow chart for selection of the study population.

This study assessed the development of children using the Denver Developmental Screening Test II (Denver II). The Denver II is a standardized screening tool designed to monitor and evaluate the overall development of preterm children from birth to 6 years of age that evaluates gross motor, fine motor, language, and personal-social development. The Denver II assessment tool is highly regarded among health care professionals due to its affordability, ease of use, and efficient application^34,35^. At the beginning of the study, we assessed the development of the 6-month-old children using the Denver II instrument, and it was determined that five families had children with suspected delayed development. Thus, these families were excluded, facilitating prospective monitoring of the remaining 259 families. There were 143 non-skipped-generation families and 116 skipped-generation families. After a period of 1 year, the monitoring process identified families that experienced changes in family and caregiver types. This resulted in a total of 247 families that remained unchanged and were available for analysis. Among these families, there were 132 non-skipped-generation families and 115 skipped-generation families. Consequently, the loss ratio for the family monitoring process was calculated to be 4.63 (Fig. 1).

### Data collection

The data obtained from the caregivers were collected using a structured questionnaire administered through face-to-face interviews. The questionnaire utilized in this research was developed by the researcher based on academic literature and related studies. Additionally, experts in the field of child development were consulted during the development process. The questionnaire comprised comprehensive items regarding the child (sex, weight status, height status, state of health, nutritional status, and electronic media use), primary caregiver (age, marital status, education level, career, state of health, and underlying diseases), and family (parent marital status, number of family members, and economic status of the family). The data were collected twice: when the child was 6 months old and 18 months old. The purpose of this questionnaire was to gather data pertaining to familial factors that may potentially impact a child's developmental process.

The content validity index of the questionnaire was assessed by a panel of five experts. These experts included a paediatrician with expertise in development and behaviour, a paediatrician specializing in mother and child health, a nursing lecturer specializing in child and family nursing, and a registered nurse specializing in family nursing. First, we calculated the item content validity index (I-CVI), which measures the validity of each item. This index is determined using the formula I-CVI = Nc/N, where Nc represents the sum of scores provided by experts who assess the items. The analysis revealed that the interview form demonstrated an I-CVI ranging from 0.6 to 1.0 for each item. The researcher implemented modifications in accordance with the recommendations provided by experts. Subsequently, the researcher employed the adjusted interview form to compute the content validity for the scale (S-CVI). The S-CVI represents the content validity of a complete interview form and is determined using the formula S-CVI = ∑I-CVI/P, where P denotes the number of questions. The study revealed that the interview form administered at 6 months of age yielded an S-CVI value of 0.82, while the interview form administered at 18 months of age yielded an S-CVI value of 0.89. Both of these values exceed the threshold of 0.8, indicating that the content validity of the interview forms was considered acceptable according to established criteria. Afterwards, the researcher used an interview form once more to conduct interviews with a population that closely resembled the sample group. It was necessary to make adjustments to ensure that the interview style was suitable for the actual study.

The development of children was assessed using Denver II tools by three registered nurses certified as early child development examiners. These nurses have been consistently conducting child development screenings using Denver II tools to date. Prior to actual data collection in the field to examine early child development, the researchers prepared the instruments and equipment and ensured that the research assistants understood the child development examination procedures to implement a standardized data collection intervention. This team of nurses was solely responsible for assessing and documenting the children's development. They did not participate in the interview. Therefore, this group of nurses was blinded to the family type (skipped-generation family or non-skipped-generation family) of the participants.

The factor of interest was the family type, which included skipped-generation families and non-skipped-generation families. Confounding factors were variables that were already known to be associated with child development during early childhood, including the child’s sex, weight status, height status, nutritional status, and electronic media use; the primary caregiver’s marital status, education level, career, and underlying diseases; the number of children receiving care, parental marital status, number of family members, and economic status of the family.

### Ethical approval

The Institutional Review Board, Office of Human Research Ethics, Khon Kaen University (HE 631,645) approved this research. This study's research was carried out in accordance with the Declaration of Helsinki. Sample groups were informed of the research's purpose, methodology, and anticipated benefits. Prior to participating, participants needed to sign an informed consent form, as participation was entirely voluntary. Members of the sample group were permitted to withdraw from the study at any time for any reason. This would have no effect on the treatment of the sample group.

### Statistical analysis

Data analysis was performed using STATA 13. We used descriptive statistics (frequencies, percentages, and the chi-square test) to analyse the baseline characteristics of children, primary caregivers, and families. We used frequencies, percentages, and 95% confidence intervals (CIs) to analyse the incidence of suspected developmental delay in early childhood at the age of 18 months and Fisher's exact test and chi-square test to compare skipped-generation and non-skipped-generation families. Subsequently, we conducted univariable analyses to examine the impact of family type on suspected developmental delay during the early childhood period at 18 months of age. We employed heterogeneity analysis to compare the incidence of suspected developmental delay between skipped-generation families and non-skipped-generation families, taking sex into consideration. The crude risk ratio (RR) was estimated by a generalized linear regression for a binary outcome (log-binomial regression) to estimate the RR with robust standard errors. Finally, we employed multivariable analyses to determine the impact of family type on suspected developmental delay during the early childhood period at 18 months of age, stratified by sex. Adjusted risk ratios were estimated by a generalized linear regression for a binary outcome (log-binomial regression) to estimate the risk ratio with robust standard errors adjusting for the sex, weight status, height status, nutritional status and electronic media use of the children, the marital status, education level, career and underlying diseases of the primary caregivers, and the number of children receiving care, parental marital status, number of family members, and economic status of the family with a p value less than or equal to 0.2. A *P* value < 0.05 was considered statistically significant.

## Results

### Baseline characteristics

At the beginning of the study, families with 6-month-old infants whose development was deemed normal using Denver II instruments were invited to participate. There were 143 non-skipped generation families and 116 skipped-generation families. After 1 year of monitoring, some families were monitored further according to the criteria and remained in the same family type. Among these families, 132 were non-skipped-generation families, while 115 were skipped-generation families.

Considering the baseline characteristics of the 6-month-old children enrolled in the study, there was no significant difference in the ratio of male to female children (proportion of male children in non-skipped-generation families versus (vs.) skipped-generation families: 48.5% and 60.9%, respectively). Children from skipped-generation families were significantly shorter than children from non-skipped-generation families (29.6% vs. 12.9%, *p* = 0.001). It was found that children from skipped-generation families were significantly more likely to be malnourished than children from non-skipped-generation families (19.1% vs. 6.8%, *p* = 0.004). However, children in skipped-generation families had a greater need for electronic media use than those in non-skipped-generation families, with statistically significant differences (non-skipped-generation families vs. skipped-generation families: 45.4% vs. 60.0%, *p* = 0.022).

Regarding the baseline characteristics of the primary caregiver, the results revealed no elderly family members as the primary caregivers in non-skipped-generation families. Skipped-generation families, on the other hand, had elderly family members as the primary caregivers. These results were significantly different (*p* < 0.001). All primary caregivers in non-skipped-generation families were found to be in good health, whereas 70.4% of the primary caregivers in skipped-generation families were found to be in good health, which was a statistically significant difference (*p* < 0.001). In addition to information about the underlying disease, it was discovered that the primary caregivers in skipped-generation families had a greater prevalence of underlying disease than primary caregivers in non-skipped-generation families (*p* < 0.001). Additionally, primary caregivers in skipped-generation families were more likely to be divorced or separated (*p* = 0.006), to have lower levels of education (*p* < 0.001) and to be unemployed (*p* < 0.001).

In regard to family type, it was discovered that skipped-generation families were significantly different from non-skipped-generation families in terms of a higher prevalence of divorced or separated parents (*p* = 0.028), a lower number of family members (*p* < 0.001), and a greater incidence of inadequate family income (*p* < 0.001) (Table 1).

**Table 1:** Baseline characteristics of the children, primary caregivers, and family.

Characteristics	Total (n = 247)	Skipped generation families (n = 115)	Non-skipped generation families (n = 132)	*p* value
*Children*				
Male sex	134 (54.3)	70 (60.9)	64 (48.5)	0.051
Underweight children	41 (16.6)	22 (19.1)	19 (14.4)	0.318
Underheight children	51 (20.6)	34 (29.6)	17 (12.9)	0.001*
Malnourished children	31 (12.6)	22 (19.1)	9 (6.8)	0.004*
First child [Birth order]	114 (46.2)	60 (52.2)	54 (40.9)	0.076
An only child, with no siblings [number of siblings]	114 (46.2)	60 (52.2)	54 (40.9)	0.076
Healthy children	247 (100)	115 (100)	132 (100)	NA
Electronic media use	129 (52.2)	69 (60.0)	60 (45.5)	0.022*
*Primary caregivers*				
Age (years)	41.41 ± 16.13	57.15 ± 7.73	27.70 ± 5.42	< 0.001*
Marital status				
Married	224 (90.7)	98 (85.2)	126 (95.5)	0.006*
Divorced/separated	23 (9.3)	17 (14.8)	6 (4.5)	
Education				
Lower than secondary school	98 (39.7)	93 (80.9)	5 (3.8)	< 0.001*
Secondary school or higher	149 (60.3)	22 (19.1)	127 (96.2)	
Career				
Unemployed	119 (48.2)	27 (23.5)	92 (69.7)	< 0.001*
Employed	128 (51.8)	88 (76.5)	40 (30.3)	
Healthy	213 (86.2)	81 (70.4)	132 (100)	< 0.001*
Underlying diseases	45 (18.2)	43 (37.4)	2 (1.5)	< 0.001*
Caregiver taking care of more than one child	92 (37.2)	39 (33.9)	53 (40.2)	0.312
*Family*				
Parent marital status				
Married	227 (91.9)	101 (87.8)	126 (95.5)	0.028*
Divorced/separated	20 (8.1)	14 (12.2)	6 (4.5)	
Number of family members				
2–4	147 (59.5)	109 (94.8)	38 (28.8)	< 0.001*
5–10	100 (40.5)	6 (5.2)	94 (71.2)	
Sufficient economic status				
Sufficient	166 (67.2)	63 (54.8)	103 (78.0)	< 0.001*
Insufficient	81 (32.8)	52 (45.2)	29 (22.0)	

*NA* data not applicable.

Data are presented as the number (%), mean ± standard deviation.

The *P* value corresponds to the independent samples *t* test or chi-square test.

*Significant at *p* < 0.05.

### Incidence of suspected developmental delay in early childhood at the age of 18 months

Forty-nine children in early childhood (18 months old) were found to have suspected developmental delay in all aspects, accounting for 19.83% of the sample (95% CI 15.05 to 25.36). Most of these children were from skipped-generation families, with 32 children accounting for 27.83% (95% CI 19.87 to 36.95) of the total sample. Children in skipped-generation families were found to have the highest level of suspected developmental delay in every aspect. There were 2 children with delays in the social aspect, representing 1.74% (95% CI 0.21 to 6.14) of the sample; 14 children with delays in the fine motor skills aspect, representing 12.17% (95% CI 6.82 to 19.58) of the sample; 22 children with delays in the language skills aspect, representing 19.13% (95% CI 12.39 to 27.52) of the sample; and 13 children with delays in the gross motor skills aspect, representing 11.30% (95% CI 6.16 to 18.55) of the sample (Table 2).

**Table 2:** Incidence of suspected developmental delay in early childhood at the age of 18 months (n = 247).

Development aspects	N	Suspected delayed development			*p* value
		n	%	95% CI	
Social skills	247	3	1.21	0.25–3.51	
Skipped-generation families	115	2	1.74	0.21–6.14	0.599
Non-skipped-generation families	132	1	0.76	0.02–4.15	
Fine motor skills	247	18	7.29	4.38–11.27	
Skipped-generation families	115	14	12.17	6.82–19.58	0.006*
Non-skipped-generation families	132	4	3.03	0.83–7.58	
Language skills	247	29	11.74	8.01–16.43	
Skipped-generation families	115	22	19.13	12.39–27.52	0.001*
Non-skipped-generation families	132	7	5.30	2.16–10.62	
Gross motor skills	247	25	10.12	6.66–14.58	
Skipped-generation families	115	13	11.30	6.16–18.55	0.565
Non-skipped-generation families	132	12	9.09	4.79–15.34	
All aspects of development	247	49	19.84	15.05–25.36	
Skipped-generation families	115	32	27.83	19.87–36.95	0.003*
Non-skipped-generation families	132	17	12.88	7.68–19.82	

The *P* value corresponds to the chi-square test or Fisher’s exact test for comparison between skipped-generation families and non-skipped-generation families.

* Significant at *p* < 0.05.

### The impact of family type on suspected developmental delay during the early childhood period at 18 months of age

In the results of the multivariable analysis pertaining to the impact of family type on suspected developmental delay during the early childhood period at 18 months of age, with consideration for sex heterogeneity, it was observed that all aspects of suspected child development delay in skipped-generation families did not show statistically significant differences when compared to non-skipped-generation families, with an aRR of 0.89 (95% CI 0.23–0.350, *p* = 0.868). When we considered each aspect of development by sex, however, we found that the number of boys raised in skipped-generation families with suspected delayed development in language skills was statistically significantly different from that of boys raised in non-skipped-generation families (aRR = 14.56, 95% CI = 1.34 to 158.34, *p* = 0.028). Additionally, the study revealed that girls who were raised by skipped-generation families exhibited significant differences in the suspected delayed development of fine motor skills compared to girls raised by non-skipped-generation families, with an aRR value of 0.06 (95% CI = 0.01 to 0.96, *p* = 0.046). The adjusted risk ratio, with robust standard errors, was calculated after accounting for various factors, including the sex, weight status, height status, nutritional status, and electronic media use of the children. Additionally, the analysis considered factors such as marital status, education level, career, and underlying diseases of the primary caregivers, as well as whether the caregiver was responsible for more than one child. Other factors taken into account included the parents’ marital status, the number of family members, and the economic status of the family, with a *p* value less than or equal to 0.2 (Table 3).

**Table 3:** Univariable and multivariable analyses of the impact of family type on suspected developmental delay during the early childhood period for 18 months by sex (n = 247).

Development aspects	Univariable analysis			Multivariable analysis		
	RR^†^	95% CI	*p* value	RR^†^	95% CI	*p* value
Social skills						
Skipped-generation families	2.30	0.21, 25.11	0.496	1.38	0.19, 10.27	0.754
Non-skipped-generation families	1.00	Reference		1.00	Reference	
Male						
Skipped-generation families	1.83	0.17, 19.86	0.620	0.96	0.15, 6.20	0.968
Non-skipped-generation families	1.00	Reference		1.00	Reference	
Female						
Skipped-generation families	–	–	NA	–	–	NA
Non-skipped-generation families	1.00	Reference		1.00	Reference	
Fine motor skills						
Skipped-generation families	4.02	1.36, 11.89	0.012*	1.17	0.07, 19.17	0.914
Non-skipped-generation families	1.00	Reference		1.00	Reference	
Male						
Skipped-generation families	3.35	0.97, 11.53	0.055	5.47	0.26, 116.23	0.276
Non-skipped-generation families	1.00	Reference		1.00	Reference	
Female						
Skipped-generation families	4.53	0.48, 42.65	0.186	0.06	0.01, 0.96	0.046*
Non-skipped-generation families	1.00	Reference		1.00	Reference	
Language skills						
Skipped-generation families	3.61	1.60, 8.15	0.002*	3.11	0.45, 21.56	0.250
Non-skipped-generation families	1.00	Reference		1.00	Reference	
Male						
Skipped-generation families	6.40	2.00, 20.53	0.002*	14.56	1.34, 158.34	0.028*
Non-skipped-generation families	1.00	Reference		1.00	Reference	
Female						
Skipped-generation families	0.38	0.04, 3.30	0.379	3.02	0.18, 51.28	0.445
Non-skipped-generation families	1.00	Reference		1.00	Reference	
Gross motor skills						
Skipped-generation families	1.24	0.59, 2.62	0.567	0.87	0.17, 4.49	0.866
Non-skipped-generation families	1.00	Reference		1.00	Reference	
Male						
Skipped-generation families	1.65	0.58, 4.67	0.349	1.59	0.12, 20.95	0.725
Non-skipped-generation families	1.00	Reference		1.00	Reference	
Female						
Skipped-generation families	0.86	0.27, 2.79	0.807	0.93	0.16, 5.49	0.939
Non-skipped-generation families	1.00	Reference		1.00	Reference	
All aspects of development						
Skipped-generation families	2.16	1.27, 3.68	0.005*	0.89	0.23, 3.50	0.868
Non-skipped-generation families	1.00	Reference		1.00	Reference	
Male						
Skipped-generation families	3.53	1.65, 7.56	0.001*	4.18	0.75, 23.38	0.103
Non-skipped-generation families	1.00	Reference		1.00	Reference	
Female						
Skipped-generation families	0.76	0.28, 2.07	0.586	0.20	0.01, 4.81	0.322
Non-skipped-generation families	1.00	Reference		1.00	Reference	

*RR* risk ratio, *CI* confidence interval, *NA* not applicable.

^†^Crude risk ratio estimated by a generalized linear regression for a binary outcome (log-binomial regression) to estimate the risk ratio with robust standard errors.

^‡^Adjusted risk ratio estimated by a generalized linear regression for a binary outcome (log-binomial regression) to estimate the risk ratio with robust standard errors adjusting for sex, weight status, height status, nutritional status, and electronic media use of the children; marital status, education level, career and underlying diseases of the primary caregivers; the number of children in the family, parental marital status, number of family members, and economic status of the family with a p value less than or equal to 0.2.

*Significant at *p* < 0.05.

## Discussion

This prospective cohort study was conducted in Khon Kaen Province. This was the first study to examine the impact of a skipped-generation family structure on the early child development of 18-month-olds using Denver II instruments to assess child development. Using multivariable analysis and adjusting for confounding variables, the results revealed that being parented by a skipped-generation family had no effect on developmental delays in children. When analysing each aspect of development by sex, it was found that boys raised in non-skipped-generation families developed significantly better language skills than those raised in skipped-generation families. However, the study revealed that girls raised by skipped-generation families had significantly better fine motor skills than those raised by non-skipped-generation families.

This study revealed that there were no significant differences in any aspect of developmental delays in children raised by skipped-generation families compared to those raised by non-skipped-generation families. However, upon conducting an analysis of each aspect of development based on sex, it was discovered that in early childhood, children raised by skipped-generation families exhibited a notable suspected delay in language development compared to children raised in non-skipped-generation families. This finding corresponds to the research conducted by Zhong J et al., which revealed a correlation between parental migration and delayed language development in children during early childhood^20^. Stimulating activities are crucial for fostering the progress of language development aspects. These activities rely on the experience and training provided by a primary caregiver^20,36–38^. In a typical family, the parents are responsible for the child's care, and a mother's care is essential for nurturing her children^8^. Many of the activities that mothers engage in while raising children encourage language development, such as reading books with their children and singing lullabies^18,39^. However, in the case of skipped-generation families, the primary caregiver is not a parent but rather a grandfather (paternal or maternal), grandmother (paternal or maternal), or great-grandparent. Due to the financial constraints within some families, grandparents or great-grandparents must serve as children’s primary caregivers instead of parents. In these families, the willingness and preparedness to nurture a child may differ from that in families in which a parent is the primary caregiver. Therefore, in skipped-generation families, the primary caregiver does not perform as many activities as the mother^20^, which may have a negative impact on the language development of the children. In addition, grandparents were found to have inappropriate responses to children's psychoemotional needs^40^, indicating that primary caregivers in skipped-generation families have distinct parenting practices and attitudes compared to families in which parents are the primary caregivers. This could potentially be an additional factor that influences the development of language in children. Regarding child sex and delayed language development, studies have shown that there is a tendency for boys to experience delayed language development in comparison to girls^41,42^. Hence, it is possible that boys raised in skipped-generation families experience delayed language development due to insufficient and inappropriate stimulation and training for language development. Moreover, this study found that in skipped-generation families, media (including television, mobile, or tablet) was employed to assist in caregiving more than in non-skipped-generation families. The primary caregivers in skipped-generation families may be unprepared to provide full-time primary care to young children. Fatigue results from raising boys, who may be more mischievous than girls. There is a tendency for caregivers to leave children alone to use media for their own convenience. This increased media exposure is another factor contributing to the delay in language development in early childhood^43^.

The results of the study found that girls raised by skipped-generation families had significantly better fine motor skills than those raised by non-skipped-generation families, and we have reservations regarding this outcome. The wide 95% confidence interval indicates that there are limited data available on girls with suspected delayed fine motor skill development, particularly for skipped-generation families in which there are three individuals, compared to non-skipped-generation families in which there is only one individual.

Interestingly, almost all of the baseline characteristic data for skipped-generation families and non-skipped-generation families were significantly different. For children, we collected data on height status, nutritional status, and the use of electronic media. For primary caregivers, we collected data on their marital status, education level, career, and underlying diseases. For families, we collected data on the marital status of the parents, the number of family members, and the family's financial situation. Numerous factors were correlated with delayed development in children. This implies that children in families with skipped generations are situated in environments that inherently pose a higher risk of experiencing developmental delays. Upon examining the crude RR without controlling for these factors, we observed a significant influence of a skipped-generation family structure on the occurrence of suspected developmental delays in fine motor skills and language skills among both boys and girls. These two developmental aspects were associated with cognitive development. This is consistent with the findings of previous studies indicating a correlation between a skipped-generation family structure and a potential delay in cognitive development among children^23,26^. This may be because families with no parents as primary caregivers were found to have an inferior home environment for children, resulting in a lack of learning materials to stimulate activities that promote skill development during early life^21,22^.

This study began collecting data on the infants when they were 6 months old. A skipped-generation family was defined as a family with no members from the parent generation, with only grandchild, grandparent, and/or great-grandparent generations as members for at least 2 months. Since Thai law permits mothers to take a 3-month maternity leave, the researchers contemplated beginning their studies on child development once the status of a skipped-generation family was established. The definition should also be applied to infants aged 6 months. If parental migration lasted for 2 months or longer, it could be interpreted as an indication that there was an agreement for the grandparents to serve as the primary caregivers for the children. Beginning the survey with 6-month-old children would help researchers determine the type of family to which the children belong. If a child was observed until the age of 18 months and if the family type persisted, this could indicate that the development of 18-month-old children is the result of parenting style. The findings of this study indicated that boys raised in skipped-generation families may exhibit potential developmental delays in their language skills. Language development is an aspect of development that is frequently identified as potential delayed, as it necessitates both training and stimulation to progress effectively. In skipped-generation families, the primary caregivers often consist of older individuals who face limitations in terms of physical strength, health conditions, attitude, knowledge, and preparedness to assume full-time responsibility for children. This is in contrast to caregivers who become parents at younger ages. In skipped-generation families, primary caregivers may engage in inappropriate caregiving practices, which may have a negative impact on the early development of children, particularly language development. It is generally recommended that elderly individuals should not be the primary caregivers for children unless it is deemed necessary. If an elderly person is to serve as a primary caregiver, it is necessary to contemplate and implement an adjustment in attitude and knowledge regarding parenting, particularly regarding an appropriate parenting style. In addition, the responsibility of parenthood should not fall solely on elderly individuals but should be shared with other family members as well.

The main strength of this study is that it is the first prospective cohort study on early childhood development conducted to evaluate and monitor child development efficiently and effectively beginning at 6 months of age using Denver II instruments. At the beginning of the study, all children participating in the study had normal development in all aspects. The study involved monitoring child development up to the age of 18 months. During this period, significant progress was observed in various aspects of development. This study is unique in its examination of the impact of a skipped-generation family structure on early childhood development. The study also conducted field data collection in a real community, encompassing both rural and urban areas where instances of parental migration are commonly observed. The study involved conducting direct interviews with primary caregivers of children and evaluating child development using Denver II tools. The evaluation was carried out by a team of professionals who possessed extensive experience, training, and certification in utilizing these tools. Nevertheless, this study was limited by its data collection, which was conducted during the COVID-19 pandemic. Certain conditions, such as family circumstances, may deviate from the norm during regular circumstances. For example, some skipped-generation families faced issues where the father and/or mother could not travel back to their hometown to visit their child due to their current residency in a pandemic area. As a result of the closure of all preschools, all of the children in this study were exclusively under the care of their families, which is not the case under normal circumstances. Since the migration decision depends on various factors, there may be unobserved factors that are correlated with both the caregiver type and child outcomes. Suggestions for future research include the study and monitoring of children with a skipped-generation family structure at other stages of life, such as school age and adolescence, with the potential benefit of a more comprehensive evaluation of child behaviour and development, as well as lingering problems. This could contribute to the accumulation of knowledge for the planning of long-term care and assistance guidelines. Children will eventually receive appropriate nurturing and continue to be a valuable resource for society.

## Conclusion

In conclusion, this study provides evidence that a skipped-generation family structure has an impact on the suspected delayed language development of boys in early childhood. The collected dataset can serve as a valuable resource for informing the planning process aimed at supporting children within skipped-generation families in early childhood. By analysing these data, policy-makers and stakeholders can develop strategies and policies that promote a more equitable and consistent distribution of economic opportunities across communities nationwide. If this endeavour proves successful, individuals belonging to the parental generation, specifically fathers and mothers, will be presented with career prospects within their local community. Furthermore, professionals in the fields of health, education, behavioural and social sciences, and psychology and other relevant stakeholders can utilize this recently acquired knowledge to adapt and enhance early childhood development models for skipped-generation families. All children will be provided with appropriate parenting and protected against potential developmental delays in the future.

## Data Availability

The datasets used and/or analysed during the current study are available from the corresponding author upon reasonable request.
